# Investigation of MicroRNA-17 Expression, Tumor Necrosis Factor-α, and Interleukin-6 Levels in Lumbar Degenerative Disc Disease: Case–Control Study

**DOI:** 10.3390/jcm14051772

**Published:** 2025-03-06

**Authors:** Luay Şerifoğlu, Müge Kopuz Álvarez Noval, Selvi Duman Bakırezer, Seda Güleç Yılmaz, Eyüp Varol, Muhittin Emre Altunrende, Ali Haluk Düzkalır, Selçuk Özdoğan

**Affiliations:** 1 Department of Neurosurgery, Umraniye Training and Research Hospital, İstanbul 34764, Turkey; 2 Department of Biochemistry, Faculty of Medicine, Yeditepe University, İstanbul 34755, Turkey; muge.kopuz@yeditepe.edu.tr; 3 Department of Basic Medical Sciences, Faculty of Medicine, Yeditepe University, İstanbul 34755, Turkey; selvi.duman@yeditepe.edu.tr; 4 Department of Medical Biology, Faculty of Medicine, Yeditepe University, İstanbul 34755, Turkey; seda.gulec@yeditepe.edu.tr; 5 Neurosurgery Clinic, Medicana Atakoy Hospital, İstanbul 34158, Turkey; dreyupvarol@gmail.com; 6 Department of Neurosurgery, Faculty of Medicine, İstinye University, İstanbul 34010, Turkey; mealtunrende@msn.com; 7 Department of Neurosurgery, Faculty of Medicine, Koc University, İstanbul 34010, Turkey; duzkalir@gmail.com; 8 Department of Neurosurgery, Camlıca Hospital, Medipol University, İstanbul 34696, Turkey; drselcukozdogan@gmail.com

**Keywords:** lumbar degenerative disc disease, microRNA-17, TNF-α, IL-6

## Abstract

**Background/Objectives:** The aim of the study is to investigate the role of microRNA-17 (miRNA-17), tumor necrosis factor-alpha (TNF-α), and interleukin-6 (IL-6) in the pathogenesis of lumbar degenerative disc disease (LDDD). The goal is to explore how miRNA-17 regulates inflammation and apoptosis within the intervertebral discs, with a particular focus on its involvement in inflammatory pathways via NF-κB signaling. This research seeks to uncover the molecular mechanisms that contribute to LDDD and its associated chronic lower back pain and disability. **Methods:** A case–control study was conducted, involving 110 patients diagnosed with LDDD and 17 healthy control individuals. Serum levels of miRNA-17, TNF-α, and IL-6 were measured using quantitative real-time PCR and enzyme-linked immunosorbent assays (ELISAs). The patients were further categorized based on the severity of their condition using the Oswestry Disability Index (ODI), which classified them into five subgroups. The correlation between miRNA-17 expression, pro-inflammatory cytokines, and disease severity was analyzed statistically. **Results:** The results demonstrated a significant downregulation of microRNA-17 in patients with LDDD compared to healthy controls. Inflammatory markers TNF-α and IL-6 were found to be significantly elevated in the patient group. A peak in inflammation and miRNA-17 expression was observed in patients with moderate to severe disability (ODI Grade 3), while inflammation levels decreased in more advanced stages of the disease (ODI Grades 4 and 5), suggesting a possible shift in disease dynamics. **Conclusions:** This study demonstrates that miRNA-17 plays a regulatory role in inflammation during the progression of LDDD, particularly through the modulation of TNF-α and IL-6 levels. The findings indicate that inflammation is most pronounced in the mid-stages of LDDD, while the later stages are characterized by structural damage rather than ongoing inflammation. These insights could help guide future therapeutic strategies aimed at targeting the molecular mechanisms underlying LDDD, potentially improving patient outcomes.

## 1. Introduction 

Lumbar degenerative disc disease (LDDD) stands out as a significant public health issue, affecting a vast portion of the adult population worldwide. Characterized by the progressive degeneration of the intervertebral discs (IVDs) in the lumbar spine, this condition is a leading cause of chronic lower back pain and disability. The pathophysiology of LDDD is multifaceted, involving a complex interplay of genetic predisposition, mechanical stresses, environmental factors, and age-related changes. Despite its prevalence and impact, the molecular mechanisms underlying the initiation and progression of LDDD remain incompletely understood, posing a challenge for developing effective therapeutic interventions.

In recent years, the field of molecular biology has made groundbreaking strides in understanding the role of microRNAs (miRNAs) in various pathological conditions. miRNAs are small, non-coding RNA molecules crucial in regulating gene expression at the post-transcriptional level. These molecules are involved in many cellular processes like cell proliferation, differentiation, apoptosis, and stress response, making them key players in disease pathogenesis. MicroRNA-17 (miRNA-17) has garnered particular attention in the context of LDDD. miRNA-17 is a member of a microRNA family involved in regulating gene expression, particularly in the context of inflammation and apoptosis [1]. In LDDD, miRNA-17 has been implicated in the modulation of inflammatory pathways. Elevated levels of miRNA-17 have been associated with pro-inflammatory cytokine production, contributing to the pathological processes of disc degeneration. miRNA-17 regulates the expression of genes that take part in cell survival and inflammation, influencing the balance between apoptosis and cell viability in the IVDs. Dysregulation of this miRNA can exacerbate inflammatory responses and increase cell death, further accelerating LDDD [2,3]. Research has shown that miRNA-17 may target specific pathways that regulate pro-inflammatory cytokines. For instance, it can modulate the signaling pathways of NF-κB, a critical regulator of inflammation. This regulation can affect the levels of cytokines like tumor necrosis factor-alpha (TNF-α) and interleukin-6 (IL-6), which are central to the inflammatory processes observed in LDDD [4]. Preliminary studies have suggested a potential link between the dysregulation of miRNA-17 and the degenerative changes observed in LDDD [5]. However, the extent and nature of this association have yet to be thoroughly investigated.

Cytokines play a key role in the inflammation response and this response elevates the circulating levels of degeneration-promoting inflammatory cytokines such as TNF-α, c-reactive protein (CRP), and IL-6 [6,7]. TNF-α is a cytokine which is secreted from white blood cells, especially macrophages. Although it has been shown to induce apoptotic or necrotic effects, it may also promote the survival and invasion of cancer cells by activating the NF-κB pathway. This pathway is stimulated by the binding of TNF-α to TNF receptor 1 (TNFR1), thereby enhancing anti-apoptotic activity [8]. TNF-α is known to play a key role in immunity, inflammation, and cancer progression [9].

Molecular biological studies confirmed the increased expression of inflammatory factors in degenerative IVDs [10,11,12]. Studies in the literature confirmed that interleukin-1β (IL-1β), IL-6, and interleukin-10 (IL-10) could mediate inflammatory response and accelerate the degeneration of IVDs [13,14]. At the same time, it has been suggested that elevated plasma concentrations of cytokines were not only associated with the degree of intervertebral disc disease but also the severity of lower back pain [15].

Recent studies have delved deeper into the immunometabolic aspects of LDDD, suggesting that inflammation is not just a byproduct but a driving force in the degeneration of IVDs. The interaction between metabolic factors and inflammation has become a focus of research, revealing potential therapeutic targets. This includes the role of adipokines (hormones produced by adipose tissue), which can exacerbate inflammation in the discs and influence the expression of miRNAs like miRNA-17 [3,4].

The aim of our study is to fill a critical gap in knowledge by conducting a comprehensive analysis of microRNA-17 expression and TNF-α and IL-6 serum levels in individuals with LDDD. This is the first study on individuals that uncovers the potential role of microRNA-17 in the pathogenesis of LDDD and compares the data of subgroups on miRNA-17, TNF-α, and IL-6 in relation to existing literature. The study’s findings are anticipated to shed light on the molecular underpinnings of disc degeneration and offer new avenues for developing targeted therapeutic strategies, potentially revolutionizing the management of these debilitating conditions.

## 2. Material and Methods

### 2.1. Study Design

This study was designed as a case–control investigation. In total, 127 participants were enrolled, comprising 110 patients diagnosed and surgery candidates with LDDD and 17 healthy control subjects. Participants were recruited from Umraniye Training and Research Hospital Department of Neurosurgery. The Umraniye Training and Research Hospital ethical Committee approved the study protocol with the number B.10.1.THK.4.34.H.GP.0.01/46 on 8 February 2024. All procedures were conducted in accordance with the ethical standards of the Declaration of Helsinki. Detailed information about the study was given to the participants and written informed consent was obtained from all of them before their inclusion.

Patients included in the study were adults aged 18 years and older. All patients had a clinically and radiologically confirmed diagnosis of LDDD and had been deemed surgical candidates by the clinical council based on magnetic resonance imaging (MRI) findings and clinical symptoms. MRI was conducted exclusively for the lumbar spine to confirm the diagnosis; however, whole-spine MRI was not performed for all patients, which is acknowledged as a potential limitation. To minimize confounding factors, patients with other spinal pathologies, including infection, tumor, fracture, or systemic diseases affecting bone metabolism (e.g., osteoporosis, rheumatoid arthritis, or diabetes), were excluded. Additionally, individuals with a prior history of lumbar spine surgery or invasive spinal procedures were not included in the study.

Healthy controls were included based on absence of lower back pain or any known spinal pathology proven with physical examination or radiodiagnostic images. They had no history of chronic diseases or long-term medication use that could affect the spine. Controls were excluded if they had any history of spinal disorders or chronic pain conditions and systemic diseases or conditions affecting the musculoskeletal system.

MicroRNA-17 expression and TNF-α and IL-6 serum levels were quantified from blood samples and using quantitative real-time polymerase chain reaction (PCR). The study proceeded with analyzing the differences in expression and serum levels between the two groups and correlating these levels with the severity of LDDD using the Oswestry Disability Index (ODI) values of patients which were taken from patients’ data archives [16]. We divided ODI severity values into 5 subgroups: 0–20 as Grade 1 (G1), 21–40 as Grade 2 (G2), 41–60 as Grade 3 (G3), 61–80 as Grade 4 (G4), and 81–100 as Grade 5 (G5).

### 2.2. Sample Collection and MicroRNA Analysis

Blood samples from the peripheral venous system were collected from all participants. All of the samples were then centrifuged at 4000 rpm (1520× *g*-force) for 15 min. The serum received from the centrifuge process was preserved at −80 °C until analysis. The serum samples were thawed and brought to room temperature to ensure optimal conditions for the planned assessments on the day of analysis.

**Isolation of miRNA and cDNA synthesis:** miRNA was extracted from the thawed serum samples utilizing the miReasy Kit (Qiagen, Germantown, MD, USA), according to the manufacturer’s guideline instructions. Then, cDNA synthesis was performed, employing the miRCURY LNA RT Kit (Qiagen, Germantown, MD, USA).

**miRNA purity measurement and concentration:** The purity of the accurate miRNA concentration levels were determined by using NanoDrop-2000 (Thermoscientific, Waltham, MA, USA). The purity of the miRNA samples was obtained by the OD260/OD280 ratio. Samples with optical density ratios results calculated to be higher than 2 were accepted as pure.

**miRNA expression analysis with real-time PCR:** The concentrations of the samples were then measured and appropriately diluted. The expression level of miRNA-17 (miRCURY hsa-miRNA-17 PCR assay, Qiagen, Germantown, MD, USA) was assessed via real-time PCR utilizing the miRCURY LNA SYBR green PCR kit (Qiagen, Germantown, MD, USA) in a Rotor Gene PCR system (Rotor-Gene Q; Qiagen, Germantown, MD, USA). The expression levels of miRNA-17 were calculated by fold change analysis using 2^−∆∆CT^ methods from Ct values obtained from a PCR instrument with RNU6 serving as the internal control for normalization.

**Enzyme-linked immunosorbent assay (ELISA):** Thawed serum samples underwent the centrifugation process at 3000 rpm (850× *g*-force) for 20 min. TNF-α and IL-6 levels in the serum were quantified using a sandwich ELISA technique with the kit (TNF-α, eBioscience, Vienna, Austria. IL-6 Assaypro, St. Charles, MO, USA) adhering to the manufacturer’s guideline instructions. A wavelength of 450 nm with an ELISA plate reader (WHYM201, Poweam Medical Co., Ltd., Nanjing, China) was used for the measurement of optical density values. The concentrations of TNF-α and IL-6 were then obtained based on the standard curve generated from the known concentrations. The results were expressed as pg/mL.

### 2.3. Statistical Analysis

IBM SPSS Statistics software version-22 (IBM Corp., Armonk, NY, USA) was eta for conducting statistical evaluations. The indicative value of statistical significance was considered as a *p*-value of less than 0.05. The comparison of the expression levels of miRNA-17 between the two groups was conducted with the Mann–Whitney *U*-test. Also, the Mann–Whitney *U*-test and ONEWAY-ANOVA test were utilized to compare TNF-α and IL-6 levels between the control group and patients.

## 3. Results

This study was composed of 17 healthy controls and 110 LDDD cases. The demographic information of the cases is displayed in Table 1.

The number of patients classified with ODI subgroups and the control group are displayed in Table 2.

**Figure 1:**
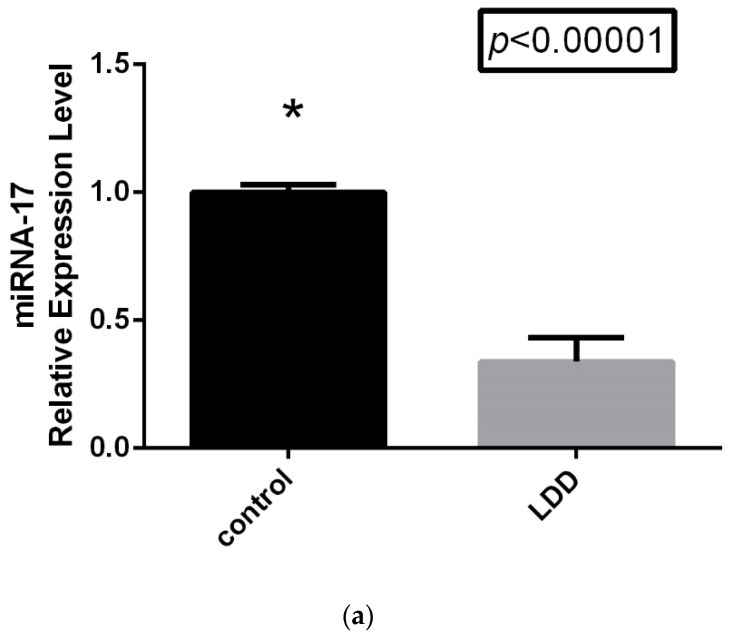

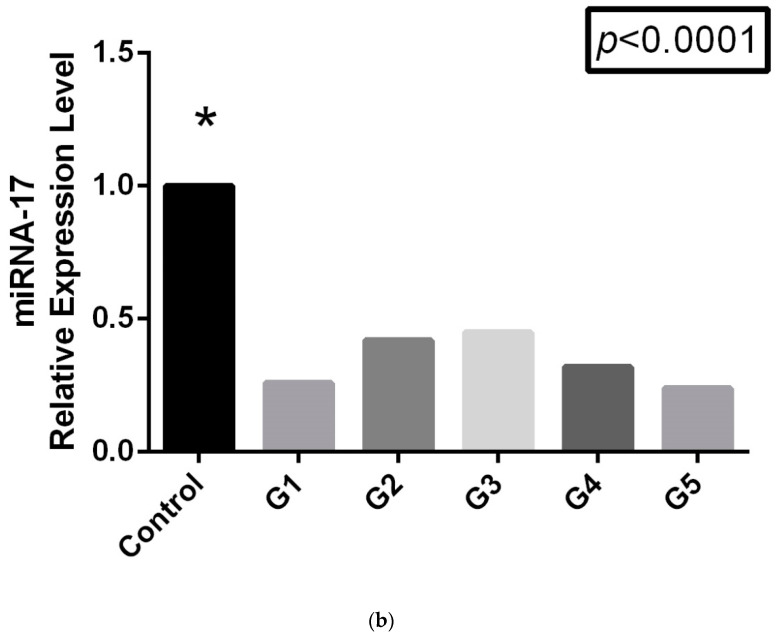
(**a**) Relative expression of circulating miRNA-17 in LDDD patient subgroups and control group (* *p* < 0.05). (**b**) Relative expression of circulating miRNA-17 in LDDD patient subgroups and control group (* *p* < 0.05).

The comparison of the relative expression levels of miRNA-17 LDDD patients and control groups is displayed in Figure 1a. As is shown in the figure, miRNA-17 expression levels were significantly downregulated in the LDDD patient group (*p* < 0.0001). The most interesting results were obtained when the LDDD subgroups were compared. As is shown in Figure 1b, miRNA-17 expression levels varied across the subgroups. However, while the control and LDDD subgroups showed a statistically significant difference, no significant difference was found between the G1 and G5 subgroups (Table 3).

**Figure 2:**
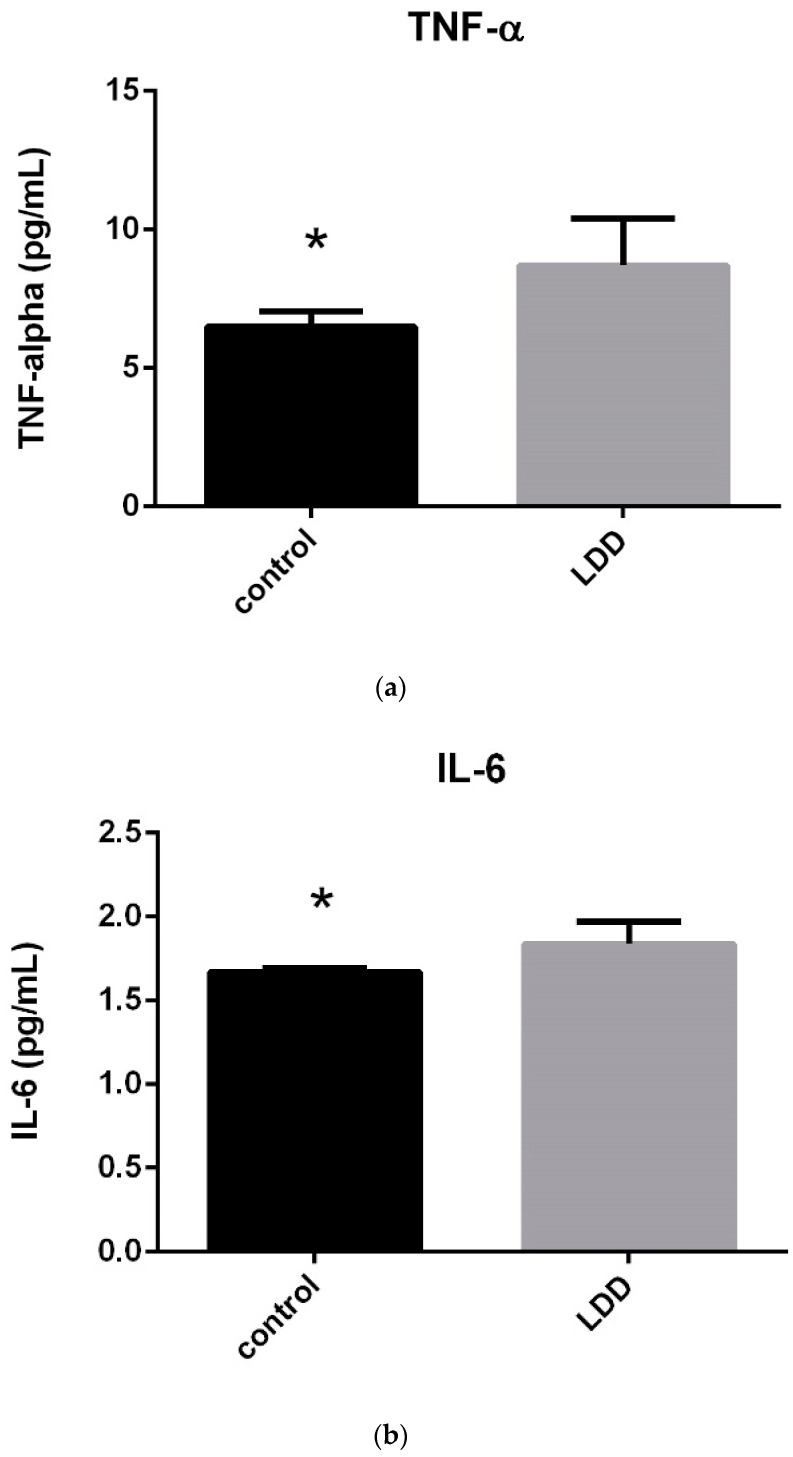
(**a**) Comparison of serum TNF-a levels in LDDD patient and control groups (* *p* < 0.05). (**b**) Comparison of serum TNF-a levels in LDDD patient and control groups (* *p* < 0.05).

As shown in Table 4, serum TNF-α and IL-6 values were significantly higher in the LDDD patient groups (*p* < 0.05) (Figure 2).

Following these results, we also conducted a subgroup analysis of LDDD patients and the control group in terms of serum TNF-α and IL-6 levels (Table 5).

All LDDD subgroups demonstrated significantly higher levels compared to the control group; however, TNF-α and IL-6 did not exhibit a progressive increase with disease severity. Moreover, serum levels of TNF-α and IL-6 were lower in the severe G5 group than in the moderate G3 group.

In light of these results, we decided to compare serum TNF-α and IL-6 levels with miRNA-17 expression levels and obtained the same histogram graphs as those obtained for the microRNA trend (Figure 2a,b).

**Figure 3:**
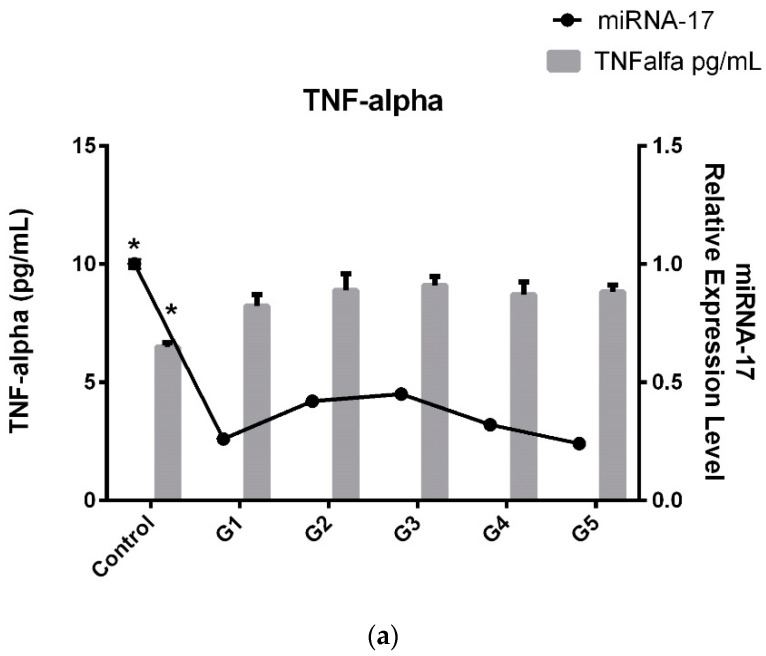

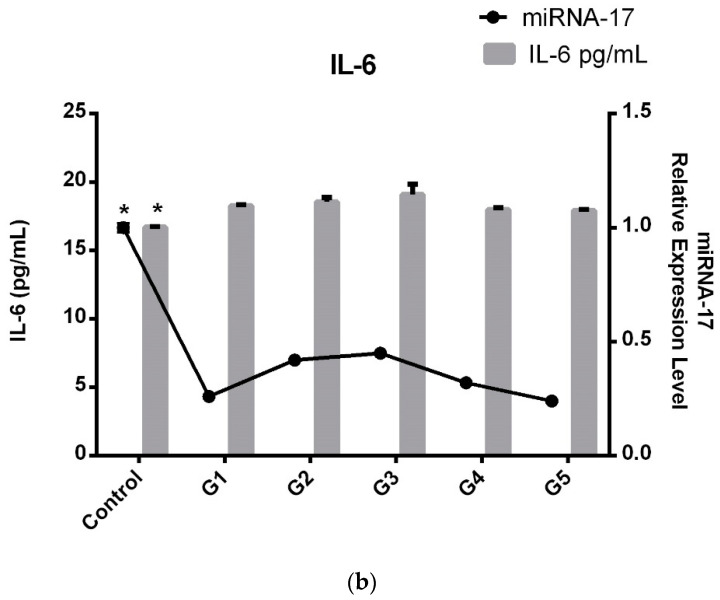
(**a**) Association between TNF-α and **relative** expression of circulating miRNA-17 in LDDD patient subgroups and control group (* *p* < 0.05). (**b**) Association between TNF-α and relative expression of circulating miRNA-17 in LDDD patient subgroups and control group (* *p* < 0.05).

Consequently, as seen in Figure 3a,b when comparing the patient subgroups, an interesting pattern emerges. TNF-α and IL-6 levels in G3 (patients with severe disability, ODI 41–60) peak, indicating that inflammation is highest in this subgroup. The increased TNF-α and IL-6 levels in G3 suggest that patients with severe disability experience a more pronounced inflammatory response, which may contribute to their higher ODI scores. Interestingly, after this peak in G3, TNF-α and IL-6 levels in G4 and G5 (patients with very severe to extreme disability) return to similar levels to those observed in G1 and G2, suggesting that inflammation may not continue to rise with greater disability severity. An interesting parallel is observed with miRNA-17 levels, which show the same trend as TNF-α and IL-6. Like inflammatory factors, miRNA-17 levels peak in the G3 subgroup, mirroring the higher inflammation observed in patients with severe disability.

In conclusion, while miRNA, TNF-α, and IL-6 levels exhibited significant variation between the patient and control groups, inflammation levels were significantly higher and miRNA-17 expression was notably downregulated. When we categorized the patients into subgroups based on their ODI scores rather than solely their LDDD diagnosis, we observed a peak in inflammation and expression in the mid G3 group. However, in the G5 subgroup, despite the disease progressing more severely and with increased pain, we did not find a decrease in expression levels or pro-inflammatory factors.

## 4. Discussion

The downregulation of miRNA-17 in LDDD patients compared to controls and its fluctuating levels across the ODI-based subgroups in this study highlight its potential role in regulating inflammation. miRNAs are small non-coding RNAs which regulate gene expression post-transcriptionally, often controlling key inflammatory pathways.

In terms of literature comparison, miRNA-17 has been reported to modulate inflammatory responses in various conditions, including inflammatory diseases and cancers [1]. For example, Quin et al. showed that miRNA-17 regulates the NF-κB pathway, a key mediator of TNF-α production, by targeting genes involved in this signaling pathway [17]. Downregulation of miRNA-17 can lead to increased activation of inflammatory genes, which aligns with the elevated TNF-α levels observed in this study.

In the context of disc degeneration, miRNA-17’s role has been less well studied, but its regulatory potential is suggested by similar findings in related degenerative diseases [18]. A study by Akhtar et al. on osteoarthritis found that the downregulation of miRNA-17 led to enhanced inflammation and cartilage degradation, similar to the degenerative processes seen in LDDD [19]. Therefore, the observed downregulation of miRNA-17 in LDDD patients may contribute to inflammatory processes by failing to suppress TNF-α and other pro-inflammatory mediators.

Interestingly, the peak in miRNA-17 levels in the G3 group observed in this study parallels the peak in TNF-α and IL-6. This suggests that miRNA-17 might be upregulated in an attempt to counteract the heightened inflammation during this stage, though this compensatory increase seems insufficient to fully suppress the inflammation. Such a dynamic regulatory role of miRNA-17 is consistent with findings in other studies, where miRNAs often exhibit context-dependent fluctuations in response to inflammatory stimuli [20].

In their study, Risbud et al. found that TNF-α and IL-6 levels were significantly higher in LDDD patients compared to healthy controls, which aligns with the existing literature on LDDD and other degenerative diseases [10]. Both TNF-α and IL-6 are well-established pro-inflammatory cytokines known to take part in the pathology of intervertebral disc degeneration. These cytokines promote matrix degradation, cell apoptosis, and pain by activating proteolytic enzymes like matrix metalloproteinases (MMPs) and inducing neuroinflammation [21].

Several studies have corroborated the finding that TNF-α plays a pivotal role in promoting inflammation and degradation in IVDs. For instance, Costachescu et al. found that TNF-α levels are elevated in degenerated disc tissues compared to non-degenerated discs, and the cytokine directly contributes to extracellular matrix breakdown and disc cell apoptosis [3]. Similarly, IL-6 has been implicated in both inflammation and pain sensitization in LDDD. Molinos et al. reported correlation between elevated IL-6 levels and pain severity in patients with degenerative disc disease, supporting the observed higher IL-6 levels in LDDD patients in this study [22].

The G3 (ODI Score 41–60) group showed the highest levels of both TNF-α and IL-6, indicating that inflammation peaks in the mid-stages of LDDD. This observation is consistent with the literature, as several studies indicate that inflammation is more intense during the early and intermediate stages of disc degeneration when disc cells and immune cells actively respond to mechanical stress and tissue damage. However, in the later stages (G4 and G5), as inflammation decreases, the focus of pathology likely shifts toward structural damage and chronic degeneration rather than acute inflammation.

The finding that inflammation peaks in the G3 group (moderate to severe disability) but declines in the more advanced stages (G4 and G5) is intriguing and aligns with some of the emerging literature on the progression of degenerative diseases. In the early to moderate stages of LDDD, inflammation is a primary driver of pain and tissue damage as immune cells infiltrate the disc and cytokines like TNF-α and IL-6 promote matrix degradation and neuroinflammation.

However, as the disease progresses, the inflammatory response may diminish due to a combination of factors, including immune cell exhaustion, fibrosis, or even the disc becoming “immunoprivileged” as a result of extensive degeneration. This phenomenon has been observed in other chronic degenerative diseases, such as osteoarthritis, where inflammation is intense in early stages but decreases as structural damage becomes the dominant feature of the disease [23,24]. This aligns with the decline in TNF-α, IL-6, and miRNA-17 levels seen in G4 and G5, where the severe disability may be more related to mechanical damage than active inflammation.

Our study provides novel insights into the pathogenesis of LDDD by highlighting the regulatory role of microRNA-17 (miRNA-17) in inflammation. While previous studies have established the association between inflammation and LDDD, our investigation uniquely explores the dynamic expression of miRNA-17 alongside TNF-α and IL-6 across different disease severity stages. This comprehensive approach differentiates our work from prior studies that often focus solely on case–control comparisons without examining disease progression in detail with subgroup analyses.

A key finding is the peak of miRNA-17, TNF-α, and IL-6 levels in the moderate to severe disability group (ODI Grade 3), which suggests a critical turning point in the inflammatory response. Unlike previous reports that broadly link inflammation to LDDD, our study identifies a potential shift in the underlying pathology from active inflammation in intermediate stages to structural degeneration in more advanced stages (ODI Grades 4 and 5). This dynamic pattern underscores the complexity of LDDD and suggests that therapeutic interventions targeting inflammation may be most effective during the mid-stages of the disease.

The observed correlation between miRNA-17 expression and inflammatory cytokines suggests that these biomarkers could play a role in guiding treatment strategies. Beyond monitoring disease progression, potential targeted pharmacological interventions include TNF-α inhibitors (e.g., Infliximab, Adalimumab), which may be beneficial in early-stage LDDD with active inflammation; IL-6 inhibitors (e.g., Tocilizumab), which have demonstrated efficacy in experimental studies on inflammatory spinal disorders; and miRNA-based therapies, which are emerging as strategies to regulate inflammation at the molecular level. Du et al. reported elevated levels of IL-6, miRNA-17, and TNF-α in related spinal disorders, including degenerative cervical myelopathy [25]. In patients with adolescent idiopathic scoliosis, Hui et al. identified differential miRNA-17 expression in bone marrow-derived Mesenchymal Stem Cells (MSCs), while Ganau et al. highlighted the role of TNF-α in inflammatory spinal pathology associated with lumbar synovial cysts [26,27]. These findings underscore the broader significance of miRNA-17, IL-6, and TNF-α in spinal pathologies and provide additional insights into their roles in LDDD pathogenesis and potential therapeutic applications.

Furthermore, the correlation between miRNA-17 expression and pro-inflammatory cytokines offers a deeper understanding of the molecular mechanisms driving LDDD. The downregulation of miRNA-17 in early and late stages, combined with its peak during intermediate stages, may indicate an adaptive but insufficient attempt to modulate inflammation. This nuanced regulation has not been previously reported and opens new avenues for research into stage-specific therapeutic strategies.

## 5. Conclusions

The results of this study are consistent with the broader literature on inflammation in degenerative disc disease. The elevated levels of TNF-α and IL-6 observed in LDDD patients align with previous findings that underscore the role of these cytokines in promoting inflammation, matrix degradation, and pain in degenerative diseases. The dynamic behavior of miRNA-17, peaking in mid-stages of the disease alongside inflammatory markers, suggests a regulatory role that is in line with its known function in modulating inflammatory pathways.

In summary, this study’s findings complement existing research by confirming the central role of inflammation in the intermediate stages of LDDD and highlighting the importance of miRNA-17 in the regulation of this process. The decline in inflammation in the later stages suggests a shift in the disease mechanism, possibly toward irreversible structural damage, which is an area that could benefit from further research to better understand therapeutic interventions for late-stage LDDD.

Lumbar degenerative disease is a multifactorial condition with significant implications for individual health and healthcare systems. The interplay between miRNA-17 and pro-inflammatory factors underscores the importance of targeted molecular therapies in managing LDDD. As research continues to unveil the complexities of this condition, it may lead to innovative treatment options that address not only symptoms but also the underlying biological processes.

Our study is the first to present a subgroup analysis that correlates miRNA-17 expression with disease severity and inflammatory markers in LDDD. This novel perspective not only enhances our understanding of LDDD progression but also highlights the potential for miRNA-17 as a biomarker for disease stage and treatment response.

## Figures and Tables

**Table 1:** Comparison of age and gender by group.

		Group		*p* Value
		LDDD (*n* = 110)	Control (*n* = 17)	
Gender	Female	20 (55.8%)	9 (35.7%)	^a^ 0.583 NS
	Male	9 (44.2%)	5 (64.3%)	
Age	Mean ± SD	46.78 ± 11.28	38.64 ± 7.11	^b^ 0.01 NS

^a^ Fisher’s Exact Test, ^b^ Mann–Whitney *U*-test. NS: nonsignificant (*p* > 0.05).

**Table 2:** The number of patients of the LDDD subgroups and control group.

Case	*n*
Control	17
Grade 1	7
Grade 2	21
Grade 3	35
Grade 4	35
Grade 5	11

*n* = number of individuals.

**Table 3:** Comparison of miRNA-17 expression levels among LDDD subgroups.

				Mean Difference		*p* Value	95% Confidence Interval
Control	vs.	G1	0.74000	0.000 *		0.7042	−0.7758
		G2	0.58000	0.000 *		0.5442	−0.6158
		G3	0.55000	0.000 *		0.5142	−0.5858
		G4	0.68000	0.000 *		0.64420	−0.7158
		G5	0.76000	0.000 *		0.7242	−0.7958
G1	vs.	G2	−0.16000	0.000 *		−0.1983	−0.1217
		G3	−0.19000	0.000 *		−0.2283	−0.1517
		G4	−0.06000	0.002 *		−0.0983	−0.0217
		G5	0.02000	0.536	(NS)	−0.0183	−0.0583

The difference between the groups was analyzed by the ANOVA test. * significant (*p* < 0.05); NS: nonsignificant (*p* > 0.05).

**Table 4:** Comparison of TNF-α and IL-6 serum levels as pg/mL between diagnosed LDDD patients and control group.

Groups	TNF-α (pg/mL)	IL-6 (pg/mL)
Controls	6.49 ± 0.16	16.70 ± 0.05
Patients	8.70 ± 0.21 *	18.38 ± 0.17 *

The difference between the groups was analyzed by the Mann–Whitney *U*-test. * significant (*p* < 0.05).

**Table 5:** TNF-α and IL-6 serum levels as pg/mL between LDDD patient subgroups and control group.

Control/Patients Groups	TNF-α (pg/mL)	IL-6 (pg/mL)
Control	6.49 ± 0.16	16.70 ± 0.05
G1	8.22 ± 0.49 *	18.30 ± 0.07 *
G2	8.63 ± 0.69 *	18.57 ± 0.30 *
G3	9.09 ± 0.37 *	19.09 ± 0.77 *
G4	8.70 ± 0.53 *	18.01 ± 0.12 *
G5	8.82 ± 0.29 *	17.93 ± 0.06

The difference between the groups was analyzed by the ANOVA test. * significant (*p* < 0.05).

## Data Availability

Dataset available on request from the authors.
